# How to Get the Full Prism Effect

**DOI:** 10.1177/2041669515599308

**Published:** 2015-08-31

**Authors:** Klaudia Pochopien, Manfred Fahle

**Affiliations:** Department of Human-Neurobiology, University of Bremen, Germany; Department of Human-Neurobiology, University of Bremen, Germany; The Henry Wellcome Laboratories for Vision Sciences, City University London, England, UK

**Keywords:** prism adaptation, sensory-motor plasticity, perceptual learning, immediate correction effect, direct effect, perspective cues

## Abstract

We investigate how the immediate correction effect decreases mispointing under prisms. Subjects performed rhythmic pointing movements under different conditions with horizontally shifting prisms. Even the first (initial) pointing error is much smaller than the prismatic shift, a phenomenon called the immediate correction effect. Knowledge about the structure of the room and of objects in the room obtained before the prisms were worn may limit the amount of the prismatic displacement perceived. We therefore compared the direct prism effect as well as prismatic adaptation with room illumination switched on versus switched off. Our 44 subjects participated in two experiments, with varying amounts of information about room structure available. The results show a direct effect corresponding to the optical power of the prisms in the dark condition, when in addition body position was slightly rotated in direction of the prismatic shift. But even in the dark, a significant immediate correction effect arises with the fixed body position. The largest immediate correction amounting to almost half of optical displacement arose in the standard condition of bright light and fixed body position.

## Introduction

In daily life, we execute many directed movements like grasping a comb, opening the door, or pointing toward an object. To grasp or to touch objects precisely requires visual information concerning the objects’ positions as well as proprioceptive information about the position of our arm (Baraduc & Wolpert, 2002).

The eye-hand coordination subsystem is without doubt one of the most important sensory-motor systems of our body (Crawford, Medendrop, & Marotta, 2004). This coordination system quasiautomatically adjusts to changes (Guan & Wade, 2000), for example, when wearing ordinary or prism glasses (Baraduc & Wolpert, 2002; Taub & Goldberg, 1973). Any discrepancy between seen and felt arm position (misalignment between spatial maps for eyes and hand) is consciously perceived and can usually be reduced within a few movements (Baraduc & Wolpert, 2002; Redding & Wallace, 1990, 1993). A prism displacing the visual field horizontally induces arm movements that initially miss the target laterally (direct effect; Guan & Wade, 2000; Harris, 1965). But with a few movements, people quickly adjust to the new visual conditions (Guan & Wade, 2000; Harris, 1965). After removing the prism glasses, participants initially miss the object in the direction opposite to the prism effect (aftereffect; Guan & Wade, 2000; Harris, 1965; Redding, Rossetti, & Wallace, 2005; Redding & Wallace, 2006). Again the mispointing decreases gradually by repeated movements and vanishes completely.

Over the last century, prism adaptation was extensively investigated (Harris, 1965; Helmholtz, 1867; Redding & Wallace, 2006), but many questions remain. For example, the immediate correction effect is only partly understood, that is, the fact that the initial effect is much smaller than to be expected: It amounts to approximately half of the optical prismatic shift (Redding, Rossetti, & Wallace, 2005).

Following the study by Rock, Goldberg, and Mack (1966), several authors (Melamed, Beckett, & Wallace, 1978; Wallace, Melamed, & Cohen, 1973) investigated the direct effect, with participants wearing prism glasses and evaluating subjective straight ahead under both totally dark and under bright light conditions. The results of Rock, Goldberg, and Mack (1966) show a difference between the bright versus dark conditions of 6° to 7° for horizontal optical displacements of 11.4°, while 8.5° for upward and 5.8° for downward directions. Explanations for the immediate correction include the ability to counteract the optical shift based on, for example, the position or size of room objects or by employing perspective cues in rooms with right angles (Rock, Goldberg, & Mack, 1966).

### Aim of the study

The present study was designed to further minimize the immediate correction effect in order to produce conditions in which the initial (pointing) error fully corresponds to the optic power of the prisms. To that aim, we tested the immediate correction effect for (a) “normal” prism experiments, (b) after removing peripheral vision (outside the prism glasses) under light conditions, (c) in the dark, and (d) after (slightly) rotating the body of participants in darkness. Our results show that under the last conditions, no immediate correction effect occurs, and the initial error closely corresponds to the optical shift.

## Methods

### Ethics Statement

The study was approved by the local ethics committee of the University of Bremen. Prior to the study, the participants were informed about the procedure which followed the Declaration of Helsinki (2008) and signed a written consent. They were free to withdraw from the study at any time, which none did.

### Participants

Forty-four participants, aged 18 to 30 years, were recruited for two experiments. Most of them were students of the University of Bremen. Two groups of 14 participants each (different individuals for each group) participated in Experiment 1 (group “adaptation in dark”: 10 females, 4 males; mean age (*M*) = 24.4 years; *SE* = 0.71; group “adaptation in light”: 10 females, 4 males; *M* = 23.2 years; *SE* = 0.74). Experiment 2 again comprised two groups, with eight participants each (group “shielded”: six females, two males; *M* = 24.8; *SE* = 0.82; group “unshielded”: six females, two males; *M* = 24.0; *SE* = 1.05). Only right-handed participants with normal or corrected-to-normal visual acuity (contact lenses only; Freiburger Visual Acuity Test; Bach, 1996), with normal stereopsis (Lang Stereo Test; Lang, 1983) and naïve to prism adaptation participated. Pupillary distance was measured with an Auto-Refractometer (NIDEK ARK-700 AUTO REF/Keratometer), to be between 54 and 64 mm for the right shifting prisms and 59 and 69 mm for the left shifting prisms.

### Experiment 1: Adaptation in Dark Versus Adaptation in Light

#### Experimental set-up

**Figure 1.**
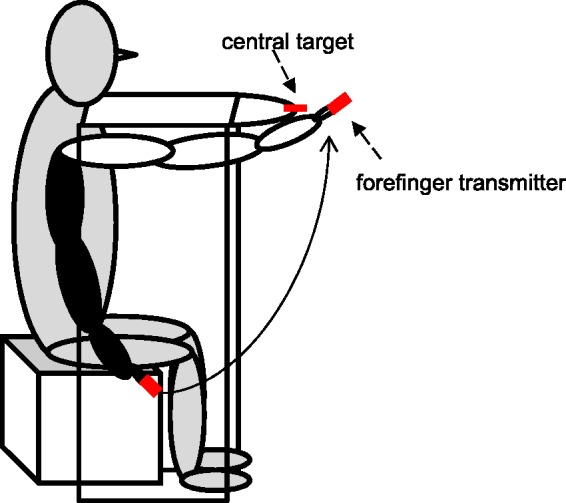
Schematic illustration of a participant seated at the table-chair while executing a pointing movement toward the central target. The black arm describes the starting position, the white arm the movement endpoint. The dashed arrow illustrates the arm movement. The red bars depict the central target (target transmitter) and the forefinger transmitter.

**Figure 2.**
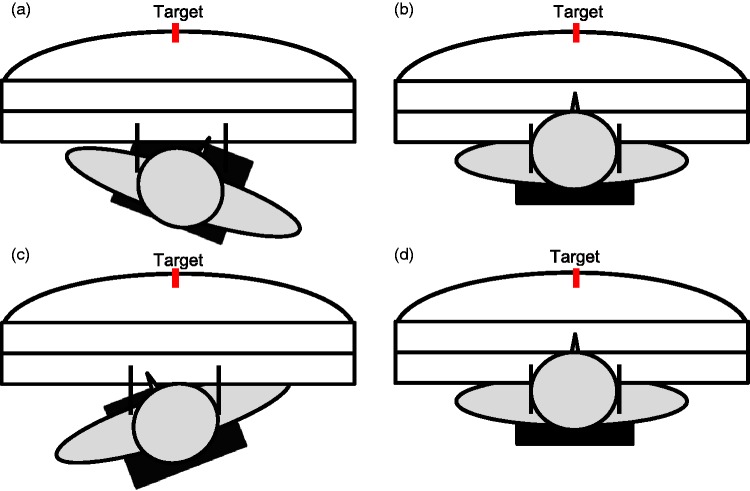
Schematic illustration of the chair positions employed: (a) prism shift and chair rotation to the right; (b) prism shift to the right and central chair position; (c) prism shift and chair rotation to the left; (d) prism shift to the left and central chair position. The red bar indicates the central target (0°).

For the adaptation in both dark and light, the apparatus consisted of a table, 99.5cm high, 110 cm wide, and 57 cm deep, with a red diode as the target (in the dark) or a red labeled stick (in the light) mounted centrally on its front side (Figures 1 and 2). Participants sat on a low chair, 43 cm high, with rotary function. The rotatability of the chair enabled a correct orientation of the participant and could be blocked to fixate its position. A chin rest served to keep target distance constant at 51 cm and to keep the central table position. For some parts of the experiment (rotated chair condition), the chin rest was not used, to enable head movement. Participants performed arm movements under the opaque table top, starting near their trunk and aiming at the visual target (Figure 1). Table depth was individually adjusted such that only 4 cm of the forefinger was visible at the end of each pointing movement, providing terminal feedback on hand position. In the dark, the right forefinger transmitter bore both an ultrasound probe and a red diode, providing visual feedback on finger localization. In the light condition, the target and the forefinger transmitter were colored red, without diodes (Figure 1).

The miniature piezo-electric transmitter on the finger emitted ultrasound which allowed to track the finger’s trajectory (Zebris Isny/Germany). We used both right- and left-shifting prism glasses (Carl Zeiss, Oberkochen/Germany). The right shifting prisms had a circular shape (Ø = 35 mm) and an optical center distance of 59 mm, whereas the left shifting prisms had oval glasses (Ø = 45 mm), with an optical center distance of 64 mm. The prismatic effect of the glasses was 30 prism diopters [cm/m], corresponding to a visual shift of 16.7°. The prism glasses had optical side shields in part of the experiments to prevent nonshifted images to reach peripheral vision.

#### Procedure

Two groups of participants performed rhythmic pointing movements in the dark (group “adaptation in dark”) or else in the light (group “adaptation in light”) toward the central target (0°) at a frequency of around 0.37 Hz (∼2.7 s per movement). In both groups, each participant was tested four times (four blocks) in the same testing order with all together 240 movements. Each block consisted of an adaptation- and a readaptation task with 30 pointing movements each. For both groups (dark and light) and two orientations of the prisms (right and left), we used two chair positions (central and eccentric), the eccentric position was to the right for right shifting prisms and to the left for left shifting prisms, yielding four conditions for each group. The three positions employed in the dark condition were central at 0°, rotated right on average at 9.3°, and rotated left at 9.9°. In the bright condition, rotation was smaller, on average 6.9° right and 4.9° left. The precise right and left positions of the chair were individually adjusted. Each participant started with the chair position rotated in the direction of the optical shift, followed by the central chair position. In Experiment 1, all measurements started with the right shifting prisms.

After being seated on the chair, participants were asked to close their eyes and to wait for further instructions. In the dark conditions, the room lights were switched off. Participants received the prism glasses while their eyes were closed and then the target was uncovered. Participants were asked to open their eyes and to rotate the chair until the target was subjectively located straight ahead of their nose, with unrestrained head position. For readaptation, the prism glasses were removed, the chair and head positions were unchanged. The results during readaptation are irrelevant for this study and not presented here, they followed the standard pattern (Figure 2).

### Experiment 2: Shielded Versus Unshielded Prisms

#### Experimental set-up

In Experiment 2, group “shielded” used prisms with an optical side shield to restrict the visual field, while group “unshielded” used prisms without a side shield. The chair was fixed at 0°, and the head was positioned in the chin rest. The experimental set-up corresponded to the previous experiment, but we only used the central chair position in a bright room, with prisms inducing a visual shift of 16.7° to the right.

#### Procedure

Participants were sitting centrally in front of the table, with their head in the chin rest and the lights switched on. Participants were asked to perform 30 rhythmic pointing movements under terminal visual feedback toward the central target (0°). For the adaptation task, they wore right shifting prism glasses, with or without optical side shields. After removal of prisms (readaptation), participants again performed 30 pointing movements toward the central target.

### Analysis

The Zebris system recorded the complete three-dimensional trajectory of the pointing movements (x-, y-, z-axis). Pointing movements were analyzed by means of a Matlab program (R2010a) developed in-house which determined the extreme of each pointing movement. The results were verified visually by the experimenters. For statistical analysis, we calculated a two-factor analysis of variance and *t* tests comparing the direct effects under different conditions.

## Results

We aimed to identify the experimental parameters responsible for the immediate correction effect. To this end, we compared (a) the direct effect for “normal” prism experiments, with (b) a condition where optical side shields eliminated the influence of peripheral, unshifted visual information, (c) adaptation in the dark, and (d) adaptation in the dark and additional body rotation.

### Experiment 1: Adaptation in Dark Versus Adaptation in Light

**Figure 3.**
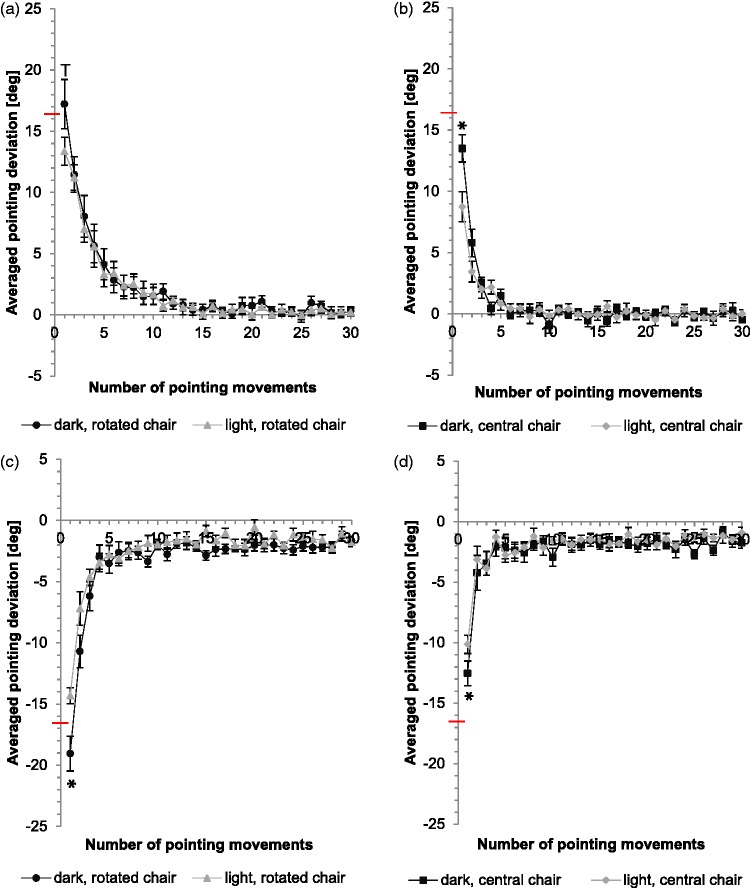
Comparison of the course of adaptive performance and direct effect between dark and light conditions for different chair positions. Black dots (rotated chair) or squares (central chair) symbolize the averaged values measured in dark and grey triangles (rotated chair) or rhombus (central chair) the averaged values measured in light. Y-axis: Horizontal deviation of the averaged pointing movements from the central target (y = 0); red horizontal line indicates the optical shift of the prisms. X-axis: Number of pointing movements performed. Error bars denote *SE*. For the initial pointing movements, significant differences between dark and light (*p* < .05) are marked with *, trends (*p* < .1) are marked with^T^ (one-sided *t* test for unpaired samples).

Figure 3 shows the averaged results for prism adaptation in the dark and in the light condition. A large initial pointing error (direct effect) in direction of the prismatic shift emerges under both conditions. The pointing error decreases substantially with further movements.

The direct effect (initial pointing error) is significantly larger in the dark than in the light condition for right shifting prisms and central chair position (*t* = 2.89, *p* = .004; one-sided *t* test for unpaired samples). The same is true for the conditions left shifting prisms and rotated as well as central chair position (*t* = −3.03, *p* = .003; *t* = −1.89, *p* = .035). The condition right shifting prisms combined with rotation to the right only yields a trend (*t* = 1.66, *p* = .054). Hence, the initial correction effect is significantly larger in light than in dark conditions (Table 1).

**Table 1.** Mean Direct Effect in Degree & Calculations of the Difference Values.

Prisms	Position	Dark [°]	Light [°]	Difference dark ↔ light
Right	Right	17.2 ± 2.0	13.4 ± 1.1	3.8 ± 2.4^T^
	Central	13.5 ± 1.1	8.7 ± 1.2	4.8 ± 1.7**
Left	Left	−19.1 ± 1.4	−14.3 ± 0.7	4.7 ± 1.3**
	Central	−12.5 ± 1.0	−10.1 ± 0.8	2.4 ± 1.5*
Difference	Right ↔ central	3.7 ± 1.7*	4.6 ± 1.3**	
Difference	Left ↔ central	6.5 ± 1.4***	4.2 ± 1.1**	

*Note.* Direct effects in degrees of visual angle. *p < .05, **p < .01, ***p < .001.

On average, the direct effect with rotated chair position in darkness slightly exceeds the optical shift of the prisms (right: 103%; left: 114%; mean: 109%). The initial pointing error with central chair amounts to only 81% in right prisms and 75% in left prisms (mean: 78%). In the light condition, the direct effect is also higher for the rotated chair condition (right: 80%; left: 86%; mean: 83%), but the effect is obviously smaller than in the dark condition. Without rotation, right prisms reach only 52% and left prisms only 61% (mean: 57%) of optical shift (mean values, see Table 2).

**Table 2.** Averaged Rotated Respectively Central Direct Effect Results (in Degree and Percent) for the Dark and the Light Condition.

Conditions	Mean direct effect [°]	Mean direct effect [%]
Dark, rotated	18.1 ± 1.2	109 ± 7.4
Dark, central	13.0 ± 0.8	78 ± 5.0
Difference rotated ↔ central (dark)	5.1 ± 1.1	31 ± 6.3
Light, rotated	13.8 ± 0.6	83 ± 3.5
Light, central	9.4 ± 0.7	57 ± 4.0
Difference rotated ↔ central (light)	4.4 ± 0.5	26 ± 3.0

*Note.* Results are tabulated in absolute values.

A two-way analysis of variance for repeated measurements was computed with prism direction and rotation as the within subjects factors and illumination as between subject factor. Please note that for statistical analysis, results for left shifting prisms were inverted. The main effect of prism direction (*F*(1, 26) = 0.73, *p* = .402) is not significant, the same is true for the interaction of prisms by group (*F*(1, 26) = 0.15, *p* = .700). The main effect of rotation is significant (*F*(1, 26) = 67.11, *p* = .000), one-sided *t* tests for paired samples as posthoc analysis show significant differences for the right shifting prisms between right and central chair position (in the dark: *t* = 2.22, *p* = .023; in the light: *t* = 3.64, *p* = .002), the same is true for the left shifting prisms (in the dark: *t* = −4.62, *p* = .000; in the light: *t* = −3.77, *p* = .001; see Table 1). However the interactions rotation by group (*F*(1, 26) = 0.37, *p* = .547), prisms by rotation (*F*(1, 26) = 0.55, *p* = .466), and prisms by rotation and by group (*F*(1, 26) = 1.06, *p* = .312) are not significant. In other words, the initial correction effect is significantly smaller for rotated than for central chair positions, under all experimental conditions.

### Experiment 2: Shielded Versus Unshielded Prisms

**Figure 4.**
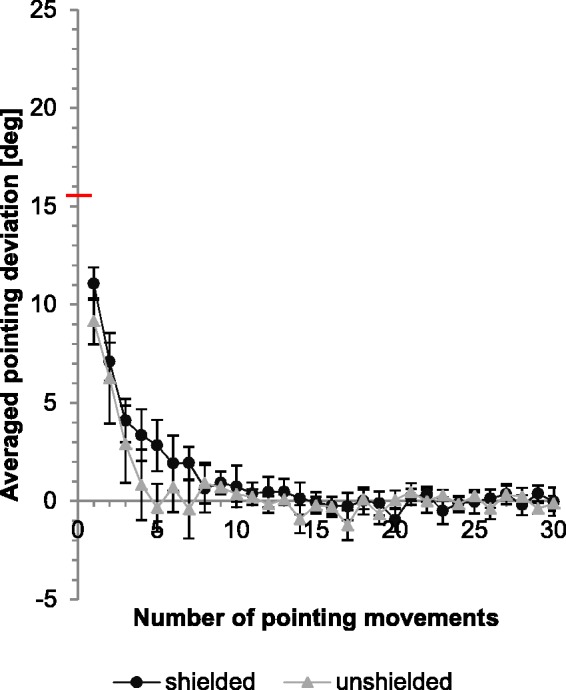
Comparison of the course of adaptive performance and direct effect between shielded and unshielded condition. Black dots indicate the average results of the “shielded” group while grey triangles symbolize those of the “unshielded” group. Y-axis: Horizontal deviation of the averaged pointing movements from the central target (y = 0); red horizontal line indicates the optical shift of the prisms. X-axis: Number of pointing movements performed. Error bars denote *SE*.

The results for the shielded (*M* = 11.1°, *SE* = 0.8) and unshielded conditions (*M* = 9.2°, *SE* = 1.2) show a large initial pointing error which decreased gradually (Figure 4).The direct effect was larger when participants wore prisms with an optical side shield with an average difference of 1.9°, but this difference fails to be significant (*t* = 1.33, *p* = .103, one-sided unpaired *t* test).

On average, the direct effect for the “shielded” group is 66% of the prismatic power, while that for the “unshielded” one is only 55%.

## Discussion

Our results on the influence of different experimental conditions show rather clear-cut results. In line with earlier studies (Melamed, Beckett, & Wallace, 1978; Rock, Goldberg, & Mack, 1966), we find that the direct prism effect, that is the deviation of the first pointing movement toward a visual target, is influenced by illumination level. Without side shields, the direct prism effect deviates from the target by only about 55% of the prismatic shift introduced by the prism glasses. The direct effect increases slightly, that is, the initial compensation effect that might be caused by knowledge of the room structure and the position of one’s own body within this room, decreases when the glasses are supplied with appropriate “side shields” thus disabling any lateral view of the room at the sides of the glasses. With this type of glasses, the direct effect increases to 66%. However, this difference fails to reach significance (*p* = .1).

The biggest increase in the direct effect of prisms onto pointing movements occurs if the pointing movements are performed in darkness, with a mean direct effect of 78% of prismatic power (right prisms: 81%; left prisms: 75%), with no significant differences (two-sided *t* test for paired samples) between right- and left-shifting prisms. This corresponds to deviations of 4.8° and 2.4° for right- and left-shifting prisms respectively, comparable to the results of Rock, Goldberg and Mack (1966). In an illuminated room, the mean direct effect only was 57% of prismatic power (right prisms: 52%; left prisms: 61%) with no significant difference between right- and left-shifting prisms.

Somewhat to our surprise, the direct effect increased (not significantly) even beyond the prismatic power when we rotated the participants to the individual’s subjective straight ahead position relative to the target in the dark after donning the prisms. The average effect was 109% (right prisms: 103%; left prisms: 114%), without significant differences between right- and left-shifting prisms. In the bright laboratory, the direct effect was on average 83% (right prisms: 80%; left prisms: 86%) without significant differences between right- and left-shifting prisms.

We fitted the adaptation curves of the individual participants by means of exponential functions of the following form, *f(x) = ae^bx^^ ^+ c.* To verify whether or not the exponential decays differ, we compared the fitted values of *b*. None of the data shown in Figures 3 and 4 yielded significant differences between the pairs of conditions compared in these figures (two-sided *t* test for unpaired samples).

A possible explanation for the effect of body rotation relies on the fact that pointing to eccentric targets without feedback, that is with the table depth extended so far that the finger cannot be seen even at the end of the movement, yields an “undershoot” of movements for eccentricities above around 8°, that is, 8° lateral target distance from the body midline for left shifting prisms (Pochopien, Stemmler, Spang, Nguelefack, & Fahle, 2013). This “undershoot” should (almost) disappear after body rotation since arm movements will be less “eccentric.” The influence of illumination, over all conditions is, on average 3.9°, while that of body position or rotation is 4.8° (Table 1), indicating a similar importance of both factors.

Our aim was to find a condition that produces a complete initial effect, that is, a pointing error corresponding to the optical power of the prisms. Therefore, we did not counterbalance between central and rotated chair positions. It is reassuring that our results for central chair position correspond closely to those of an earlier study on prism adaptation in the dark without chair rotation (Rock, Goldberg, & Mack, 1966).

From our results, we conclude that the initial effect of prisms is (at least) as large as to be expected from the optical quality of the prisms when tested in an adequate way. But under standard experimental conditions, information and knowledge about room structure in general (such as perspective) and about the room at hand will decrease the size of the mispointing, to about half of prismatic power as does the fact that eccentric positions in general tend to be underestimated (Parise, Spence, & Ernst, 2012). This finding agrees with assumptions of Rock, Goldberg, and Mack (1966). Hence we conclude that in the future, we do not have to worry about the fact that the direct effect is so much smaller than the optic shift induced by the prismatic glasses, but we must be aware that the size of the direct effect and hence our results may rely on a number of factors such as the exact type of glasses employed (sunglass-type vs. diver-type), the structure of the experimental chamber, and body orientation or target eccentricity.
